# Minimally Invasive Esophagectomy for Esophageal Cancer: Current Evidence and Future Perspectives

**DOI:** 10.1002/ags3.70112

**Published:** 2025-10-16

**Authors:** Hirotaka Konishi, Hiroyuki Inoue, Hitoshi Fujiwara, Atsushi Shiozaki

**Affiliations:** ^1^ Division of Digestive Surgery, Department of Surgery Kyoto Prefectural University of Medicine Kyoto Japan

**Keywords:** esophageal cancer, mediastinoscopy, minimally invasive esophagectomy, robot‐assisted laparoscopy, thoracoscopy

## Abstract

**Aim:**

Esophageal cancer is a highly aggressive malignancy with regional variations in histological subtypes. Adenocarcinoma predominates in Western countries, whereas squamous cell carcinoma is more common in Asia. Despite advances in multimodal therapy, esophagectomy remains the cornerstone of curative treatment, and the development of various minimally invasive esophagectomies (MIE) has been promoted to reduce invasiveness and complications. The status of the MIE has been outlined.

**Methods:**

A comprehensive literature review was conducted using PubMed/MEDLINE to identify relevant studies on MIE published up to June 2025. The search focused on thoracoscopic, robot‐assisted, and mediastinoscopic approaches, with an emphasis on randomized trials and high‐quality comparative studies.

**Results:**

Thoracoscopic MIE, especially in the prone position, demonstrated reduced pulmonary complications and shorter recovery times than open surgery, as supported by randomized trials such as TIME, MIRO, and MONET. Robot‐assisted MIE (RAMIE) further enhances lymph node dissection and improves preservation of the recurrent laryngeal nerve, as demonstrated in trials such as REVATE and RAMIE. Mediastinoscopic esophagectomy via the transcervical and transhiatal approaches is emerging as a promising alternative for high‐risk patients, offering favorable perioperative outcomes with reduced pulmonary complications. Further evaluation is required to determine the efficacy of lymph node dissection and the risk of recurrent laryngeal nerve damage.

**Conclusion:**

MIE, including the thoracoscopic, robot‐assisted, and mediastinoscopic approaches, is evolving into an effective and less invasive alternative to open surgery. Future research should focus on conducting standardized, multicenter trials to establish optimal surgical strategies based on tumor characteristics and patient‐specific factors.

## Abstract 2

The development of various minimally invasive esophagectomies (MIE) has been outlined in this review article. MIE, including the thoracoscopic, robot‐assisted, and mediastinoscopic approaches, is evolving into an effective and less invasive alternative to open surgery. Future research should focus on conducting standardized, multicenter trials to establish optimal surgical strategies based on tumor characteristics and patient‐specific factors.

## 1 Introduction

Esophageal cancer is one of the most aggressive malignancies with a poor global prognosis [1], and its histological subtypes vary by region. Adenocarcinomas of the lower esophagus account for many cases in the United States and European countries, whereas squamous cell carcinoma (SCC) is more prevalent in Asian countries [2]. Although multidisciplinary approaches, including surgery, chemotherapy, and radiotherapy, have been developed for the treatment of esophageal cancer, conventional esophagectomy remains the definitive curative option [3]. Esophagectomy is the most invasive oncologic procedure and has historically been associated with high rates of pulmonary complications, vocal cord palsy, and prolonged recovery [4]. Surgical techniques, particularly lymphadenectomy strategies, also vary by region due to differences in histological subtypes. Recent advancements in early diagnostic technologies have expanded the indications for surgical intervention; nonetheless, concerns regarding postoperative complications and quality of life remain. Advancements in optics, stapling devices, and robotic articulation have enabled the implementation of laparoscopic and robotic approaches. Given the invasiveness of conventional open surgery, minimally invasive esophagectomy (MIE) is increasingly adopted to reduce the physical burden on patients.

Various surgical procedures for MIE have been reported [5]. Thoracoscopic esophagectomy via the right transthoracic approach in the prone position has become common [6], and the number of reports on robot‐assisted MIE (RAMIE) is steadily increasing [7]. However, a growing number of reports, particularly from Asian countries, have described non‐thoracic MIE approaches that combine transcervical and transhiatal procedures [8]. As a result, the clinical question has shifted from “Is MIE feasible?” to “Which minimally invasive modality offers the best oncological outcomes with the fewest complications?” This article reviews the different types of minimally invasive surgeries for esophageal cancer, highlighting their advantages and disadvantages, clinical outcomes, and future challenges.

## 2 Materials and Methods

A systematic search of the scientific literature was conducted using PubMed/MEDLINE to identify all relevant articles on MIE for esophageal cancer published up to June 2025. The primary search terms included “minimally invasive esophagectomy,” “thoracoscopic esophagectomy,” “robot‐assisted esophagectomy,” and “mediastinoscopic esophagectomy,” including transcervical and transhiatal approaches. The reference lists of the selected articles were also reviewed to identify additional relevant studies.

Articles published in non‐English languages were excluded. All major available publications were reviewed to identify those focusing on MIE. Smaller‐scale studies were also included if meaningful data relevant to MIE were provided. We excluded case reports involving only a few patients and studies focused on noncurative procedures, such as salvage or palliative surgery.

The search focused on thoracoscopic, robot‐assisted, and mediastinoscopic approaches, with an emphasis on randomized trials and high‐quality comparative studies. On the other hand, there are few such studies regarding mediastinoscopic esophagectomy. Ultimately, this review article became a narrative review, taking into account the quality of studies, particularly those related to mediastinoscopic esophagectomy.

## 3 Results

### 3.1 Evolution of Techniques in Thoracoscopic and Laparoscopic Esophagectomy

At the beginning of MIE, thoracoscopic and laparoscopic surgeries were increasingly performed since the 1990s to reduce the surgical risk and burden on patients with esophageal cancer [9]. Cuschieri reported on endoscopic‐assisted esophagectomy performed in the left lateral decubitus or prone position, noting patients converted to open thoracotomy and associated postoperative complications. They concluded that the prone position offered technical advantages and was associated with fewer respiratory complications. Since the 2000s, thoracoscopic MIE in the prone position has become more common, as numerous reports have demonstrated favorable outcomes [10].

### 3.2 MIE via Transthoracic Approaches

Since the initial adoption of MIE, numerous studies, including meta‐analyses and systematic reviews, have reported the outcomes of thoracoscopic and/or laparoscopic esophagectomy for esophageal cancer, comparing these approaches to conventional open esophagectomy in terms of surgical results, postoperative complications, and long‐term prognosis [11, 12, 13, 14] (Table 1). Biere et al. [15] reported the results of the TIME trial, a randomized controlled trial comparing MIE with open esophagectomy. This study enrolled 56 patients in the open surgery group and 59 patients in the MIE group. The incidence of in‐hospital pulmonary infections was significantly lower in the MIE group (12%) compared to the open surgery group (34%) (*p* = 0.005), suggesting that MIE offers short‐term benefits to patients with resectable esophageal cancer. However, long‐term outcomes showed no significant difference in the overall survival (50.5% in MIE vs. 40.4% in open esophagectomy, *p* = 0.207) [16].

**TABLE 1:** Summary of major randomized controlled trials comparing open and minimally invasive esophagectomy.

Trial	Study design	Sample size	Comparison	Short‐term outcomes	Long‐term outcomes
TIME trial [15, 16]	Randomized Controlled (Europe)	Open: 56 MIE: 59	Open esophagectomy vs. totally MIE	Pulmonary infection (*p* = 0.005): 34 (open) vs. 12 (MIE) %	3‐year OS (*p* = 0.207): 40 (open) vs. 51.5 (MIE) %
MIRO trial [14, 17]	Multicenter Randomized Phase III (France)	Open: 104 Hybrid: 103	Open esophagectomy vs. Hybrid (lap abdominal + open thoracic)	Major complications (*p* < 0.001): 64 (open) vs. 36 (hybrid) % Pulmonary complications: 30 (open) vs. 18 (hybrid) %	5‐year OS (Not significant): 40 (open) vs. 60 (hybrid) %
MONET trial [18, 19] (JCOG1409)	Randomized Phase III (Japan)	Open: 150 Thoracoscopic: 150	Open esophagectomy vs. thoracoscopic	R0 resection: 90 (open) vs. 95.3% (thoracoscopy) % Perioperative morbidity: equivalent Postoperative respiratory dysfunction: less in thoracoscopy	3‐year OS (non‐inferiority): 70.9 (open) vs. 82 (thoracoscopy) % 3‐year PFS (non‐inferiority): 61.9 (open) vs. 72.9 (thoracoscopy) %

Abbreviation: MIE, minimally invasive esophagectomy.

Briez et al. [14] conducted the MIRO trial, a multicenter randomized phase III trial that compared open esophagectomy with laparoscopically assisted (hybrid) esophagectomy. They hypothesized that the laparoscopic abdominal approach in esophageal cancer surgery would decrease the major postoperative complication rate owing to reduced surgical trauma. In this study, 103 patients underwent hybrid procedures (open right thoracotomy with laparoscopic gastric mobilization) and 104 underwent fully open surgery [17]. The hybrid group had significantly fewer major intraoperative and postoperative complications (36% vs. 64%, OR: 0.31, *p* < 0.001), and fewer major pulmonary complications (18% vs. 30%, OR: 0.50). Although the five‐year overall and disease‐free survival rates were numerically higher in the hybrid group (60% vs. 40% and 53% vs. 43%, respectively), these differences were not statistically significant. These findings suggest that minimally invasive abdominal procedures may reduce the risk of postoperative complications such as pneumonia.

Studies from Western countries mainly included patients with adenocarcinoma located in the lower third of the esophagus, which is more common in Europe. Kataoka et al. [18] conducted the MONET trial (JCOG1409), a randomized phase III study in Japan, comparing thoracoscopic and open transthoracic esophagectomy for thoracic esophageal cancer. A total of 300 patients were randomized to undergo either thoracoscopic or open surgery to demonstrate the non‐inferiority of thoracoscopic esophagectomy in terms of overall survival. Although detailed results have yet to be published, the trial was terminated early based on recommendations from the Data and Safety Monitoring Committee, and preliminary results were presented at the 2024 ASCO Gastrointestinal Cancer Symposium [19]. Three‐year overall survival and relapse‐free survival rates were superior in the thoracoscopic group (82% vs. 70.9% and 72.9% vs. 61.9%, respectively). Postoperative morbidity rates were comparable; however, respiratory dysfunction was less frequent in the thoracoscopic group. This trial is considered the first to confirm the non‐inferiority of thoracoscopic esophagectomy to open surgery in terms of survival outcomes for thoracic esophageal cancer. These results suggest that thoracoscopic MIE should be the standard surgical procedure for thoracic esophageal cancer.

Collectively, the benefits of thoracoscopic esophagectomy, particularly reduced respiratory complications, have been supported by high‐quality randomized controlled trials and comparative studies [12, 13, 14]. However, several meta‐analyses and large‐scale database studies identified technical challenges and drawbacks. Takeuchi et al. [20] analyzed the short‐term outcomes of MIE versus open esophagectomy using a nationwide Japanese database with propensity score matching. They found that, while blood loss was lower in the MIE group and the need for postoperative mechanical ventilation over 48 h was reduced, the operative time was longer. The 30‐day reoperation rate was significantly higher in the MIE group (7.0% vs. 5.3%, *p* = 0.004). Conversely, the 30‐day (0.9% vs. 1.1%) and operative (2.5% vs. 2.8%) mortality rates did not differ significantly. These findings are consistent with those of other reports evaluating the MIE in thoracic esophageal cancer [11, 13, 14, 16].

### 3.3 Robot‐Assisted MIE via Transthoracic Approaches

RAMIE has been introduced to overcome the limitations of conventional open esophagectomy and serves as an alternative to thoracoscopic MIE, and promising results have been reported so far [7] (Table 2). Severe postoperative complications such as anastomotic leakage and pneumonia have been shown to adversely affect long‐term survival [36, 37, 38]. RAMIE is expected to reduce the incidence of such complications and potentially improve prognosis owing to its articulated instruments, tremor‐filtering systems, and enhanced magnified visualization [21, 22]. Kernstine et al. [23] first reported robot‐assisted esophagectomy in 2004, performing a thoracic esophagectomy with three‐field lymphadenectomy in a modified left‐lateral‐to‐prone position for the thoracic phase and a supine position for the abdominal phase. Since then, numerous comparative studies and review articles have been published assessing the outcomes of RAMIE, thoracoscopic MIE, and open esophagectomy [24, 25], and several large‐scale analyses using nationwide cohorts have also been conducted [26, 27, 28, 29, 30, 31]. In many of these reports, RAMIE has been associated with shorter hospital stays and reduced conversion rates to open surgery. Additionally, an increased number of lymph nodes, particularly those around the recurrent laryngeal nerve (RLN), have been dissected using this approach. Although operative mortality rates have been reported to be similar across surgical modalities, reports have varied regarding operative time and incidence of major postoperative complications.

**TABLE 2:** Summary of RAMIE outcomes and key comparative clinical trials.

Study/source	Design	Comparison	Key findings (vs. MIE)
General findings [7, 21, 22, 23, 24, 25, 26, 27, 28, 29, 30, 31]	Review‐based	RAMIE vs. MIE/Open	Improve: number of RLN lymph node harvest Deteriorate: conversion rate, hospital stay Equivalent: mortality Mixed results: operative time, complications
Perry et al. [25]	Meta‐analysis	RAMIE vs. MIE	Improve: number of lymph node harvests, blood loss, pulmonary complications, hospital stay Deteriorate: operative time, anastomotic leakage RAMIE is a safe and feasible alternative, but significant heterogeneity
ROBOT‐2 trial [32]	RCT (ongoing) (multicenter)	RAMIE vs. MIE (esophageal adenocarcinoma)	Primary endpoint: number of dissected abdominal and mediastinal lymph nodes expected to clarify oncologic efficacy of RAMIE
REVATE trial [33, 34]	RCT (multicenter)	RAMIE vs. MIE (103 vs. 100, ESCC)	Improve: mediastinal lymph node harvest successful left RLN dissection (88.3 (RAMIE) vs. 69 (MIE) %, *p* < 0.001) permanent RLN palsy (5.8 (RAMIE) vs. 20 (MIE) %, *p* = 0.003) RAMIE enables safer and more effective left RLN lymphadenectomy
RAMIE trial [35]	RCT (multicenter)	RAMIE vs. MIE (181 vs. 177, ESCC)	Improve: number of thoracic/left RLN lymph node harvest (*p* = 0.016/0.001) operative time (*p* < 0.001) Equivalent: blood loss, R0 rate, complications RAMIE is a safe and oncologically effective alternative with favorable perioperative outcomes

Abbreviations: MIE, minimally invasive esophagectomy; RAMIE, robot‐assisted MIE; RCT; randomized controlled trial; RLN, recurrent laryngeal nerve.

Perry et al. [25] conducted a meta‐analysis of these studies to compare the short‐term outcomes between conventional MIE and RAMIE. Their findings suggest that RAMIE is a safe and feasible alternative to conventional MIE, demonstrating advantages in terms of blood loss, lymph node yield, pulmonary complications, and length of hospital stay. Conversely, conventional MIE has shown benefits, including shorter operative times and a lower rate of anastomotic leakage. The authors noted significant heterogeneity among the included studies and emphasized the need for further high‐quality prospective studies to validate these findings.

Several randomized controlled trials have compared the outcomes of thoracoscopic MIE and RAMIE. The ROBOT‐2 trial was a randomized controlled trial in Europe that compared conventional MIE with RAMIE for the treatment of esophageal adenocarcinoma [32]. This trial is ongoing, and its primary endpoint is the total number of dissected lymph nodes in the abdominal and mediastinal regions. The results of this comparison are expected to clarify whether RAMIE offers superior oncological clearance. Chao et al. [33] conducted a multicenter, open‐label, randomized controlled trial, REVATE, designed to assess the superiority of RAMIE over thoracoscopic MIE in performing lymphadenectomy along the left RLN in patients with esophageal squamous cell carcinoma (ESCC). This trial, reported in 2024 [34], included 103 patients in the RAMIE group and 100 patients in the MIE group from three Asian centers. Successful left RLN lymphadenectomy was achieved in 88.3% of patients in the RAMIE group, compared to 69% in the MIE group (*p* < 0.001). Furthermore, the incidence of left RLN palsy was significantly lower in the RAMIE group (20.4% vs. 34%; *p* = 0.029). The number of dissected mediastinal lymph nodes was also higher in the RAMIE group (median: 16 vs. 14; *p* = 0.035). Therefore, the authors concluded that RAMIE enabled a more effective and safer left RLN lymphadenectomy. Yang et al. [35] conducted a prospective randomized controlled trial, referred to as the RAMIE trial, across six high‐volume centers in China to compare the perioperative and long‐term outcomes of RAMIE and conventional MIE in patients with ESCC. In the RAMIE group, significantly shorter operation times (*p* < 0.001) and improved lymph node dissection efficiency were observed in the thoracic (*p* = 0.016) and left RLN (*p* = 0.001) regions. No significant differences were found in blood loss, conversion rates, R0 resection rates, or overall complication rates between groups. The investigators concluded that RAMIE is a safe and oncologically effective alternative to conventional MIE, offering favorable short‐ and mid‐term outcomes. As a result, RAMIE is gradually replacing conventional MIE.

### 3.4 Mediastinoscopic Esophagectomy via Transcervical and Transhiatal Approaches

MIE via the transthoracic approach is the mainstream radical procedure for the treatment of esophageal cancer. Esophagectomy via a mediastinoscopic approach has been developed as a minimally invasive radical option (Table 3). Transhiatal esophagectomy without the transthoracic approach was first reported by Orringer and Sloan [45]. Although this procedure avoids thoracotomy, it involves blunt dissection and does not allow for mediastinal lymphadenectomy. Subsequently, Bumm et al. [46] and Tangoku et al. [47] reported on mediastinoscopic esophagectomy with mediastinal lymph node dissection using specialized equipment. This procedure is an alternative MIE technique that avoids the transthoracic approach and reduces the risk of pulmonary complications. However, limited operative space and insufficient mediastinal visibility make it a less radical option.

**TABLE 3:** Summary of mediastinoscopic esophagectomy.

Author	Design	Comparison	Key findings (vs. thoracoscopy)
Fang et al. [8]	Meta‐analysis	Mediastinoscopic vs. thoracoscopic	Improve: operative time, blood loss, pulmonary complication, hospital stay Deteriorate: number of lymph node harvest, RLN injury Equivalent: long‐term survival
Wang et al. [39]	Meta‐analysis	Mediastinoscopic vs. thoracoscopic	Improve: operative time, pulmonary complication, hospital stay Deteriorate: number of lymph node harvest, RLN injury Equivalent: blood loss, postoperative complication, mortality
Dabsha et al. [40]	Systematic review	Pooled data	Improve: operative time, blood loss, postoperative morbidity Equivalent: oncological safety, lymph node dissection
Zhang et al. [41]	Observation study (single center)	Mediastinoscopic vs. thoracoscopic (38 vs. 80)	Improve: postoperative pneumonia Deteriorate: RLN injury Equivalent: number of mediastinal lymph node harvests, long‐term survival
Shi et al. [42]	RCT (multicenter)	Mediastinoscopic vs. thoracoscopic (100 vs. 100)	Improve: operative time, blood loss, postoperative pneumonia, hospital stay Deteriorate: number of lymph node harvests Equivalent: RLN injury, anastomotic leakage
Yin et al. [43]	RCT (single center)	Mediastinoscopic vs. thoracoscopic (40 vs. 40)	Improve: operative time, number of left RLN lymph node harvests, postoperative pain score, pulmonary complication, hospital stay Equivalent: blood loss, total number of mediastinal lymph nodes
Vercoulen et al. [44]	Prospective cohort study (single center)	Mediastinoscopic: 75	Improve: pulmonary complication (16%) Deteriorate: temporary RLN palsy (44%)

Abbreviations: RCT, randomized controlled trial; RLN, recurrent laryngeal nerve.

To overcome these limitations, a new mediastinoscopic esophagectomy generally using the transcervical and transhiatal approaches has been reported for the first time from Japan [48, 49]. This procedure offers advantages in terms of operative time and pulmonary complications compared with conventional transthoracic MIE, as it does not require a transthoracic approach, one‐lung ventilation, or a change in patient position from the supine position. The accuracy of mediastinal lymphadenectomy has improved, and an increasing number of reports suggest that mediastinoscopic esophagectomy is being performed as a curative procedure comparable to transthoracic esophagectomy [50, 51, 52, 53]. These studies reported that mediastinoscopic esophagectomy was performed safely and was superior to open or thoracoscopic esophagectomy in terms of shorter operative time and fewer postoperative respiratory complications.

It should be noted that transhiatal procedures vary slightly depending on the institution and reports, compared to transthoracic approaches, which are becoming more standardized. The transhiatal approach was performed using a hand‐assisted laparoscopic surgery technique, which has also been used for abdominal manipulation during open and thoracoscopic esophagectomy [51, 54]. Mori et al. [55] originally performed a transhiatal procedure using a robot‐assisted laparoscopic technique. More recently, a transhiatal approach using a completely laparoscopic technique has also been reported [53, 56].

Conversely, the transcervical procedure was initially reported to use a single‐port technique through a left cervical incision only [51, 52, 53, 54, 55]. Fujiwara et al. [51] reported that upper and middle mediastinal lymph node dissection up to the carina can generally be performed safely and reliably using the left cervical approach alone. However, some reports have indicated that the right cervical approach is particularly useful for dissection of the upper mediastinum on the right side of the esophagus, and the number of reports describing the bilateral transcervical approach is increasing [56, 57, 58].

Although mediastinoscopic esophagectomy can be completed using a laparoscopic technique [51, 52, 53, 54, 55], some reports have suggested that robot‐assisted mediastinoscopy is useful for precise and stable manipulation of the narrow and deep mediastinum. Yoshimura et al. performed radical mediastinoscopic esophagectomy using robot‐assisted laparoscopy via the transhiatal approach and concluded that robot‐assisted mediastinoscopic esophagectomy offers short‐term benefits compared with transthoracic esophagectomy [59]. Fujita et al. reported robot‐assisted transcervical technique using a bilateral cervical approach. They safely performed robot‐assisted mediastinoscopic esophagectomy on both sides of the neck and concluded that this technique may reduce the incidence of RLN palsy [60]. Hadzijusufovic et al. conducted a trial using the da Vinci SP system for transcervical approach [61]. They reported that mediastinal dissection using the da Vinci SP system was effective. Nonetheless, its clinical utility and reproducibility require further investigation.

Several meta‐analyses and systematic reviews have been reported [8, 39, 40, 41]. These studies suggest that mediastinoscopic esophagectomy is a promising MIE technique and a feasible alternative to thoracoscopic MIE. It has shown advantages in reducing the operative time, blood loss, and pulmonary complications [8, 39, 41]. However, a relatively high incidence of RLN palsy has been reported [8, 39, 41], and the number of dissected lymph nodes may be lower than that in transthoracic MIE [8, 39]. Further randomized studies are required to evaluate the oncological efficacy and long‐term outcomes of mediastinoscopic esophagectomy.

There are still fewer comprehensive studies, three prospective randomized or cohort studies have been conducted in China and the Netherlands [42, 43, 44]. Shi et al. prospectively compared mediastinoscopic and thoracoscopic esophagectomy. They concluded that the operative time, blood loss, postoperative pneumonia, and hospital stay were significantly improved, but the number of dissected lymph nodes was decreased in mediastinoscopic esophagectomy [42]. Yin et al. reported that postoperative pulmonary complications were reduced, and that mediastinoscopic esophagectomy may also be suitable for patients with chronic lung disease, poor pulmonary function, or those who are not good candidates for thoracic surgery [43]. Vercoulen et al. conducted a prospective cohort study on transcervical MIE at a Dutch institution. They concluded that postoperative pulmonary complications were reduced, although the incidence of temporary RLN palsy increased [44].

## 4 Discussion

MIE has undergone significant evolution over the past three decades, with the aim of reducing surgical trauma and improving perioperative outcomes without compromising oncological efficacy [5]. The widespread adoption of thoracoscopic and laparoscopic techniques, followed by the emergence of robot‐assisted and mediastinoscopic approaches, reflects dynamic progress in this field.

Among the various MIE techniques, thoracoscopic esophagectomy in the prone position has become the standard approach for lower esophageal adenocarcinoma, particularly in Western countries [11, 13]. Randomized trials such as TIME [15, 16] and MIRO [14, 17] have demonstrated that MIE and hybrid procedures are associated with significantly fewer pulmonary complications and shorter recovery times than open surgery, although the survival benefits remain unclear. In Asia, where mid‐to‐upper esophageal SCC is more common, the MONET trial (JCOG1409) provided robust evidence of the non‐inferiority of thoracoscopic esophagectomy in terms of survival, along with reduced postoperative respiratory dysfunction [18, 19]. These findings reinforce the growing consensus that thoracoscopic MIE can achieve oncologic outcomes comparable to those of open surgery while reducing perioperative morbidity.

Robot‐assisted MIE has further enhanced the technical precision of esophagectomy, particularly in lymphadenectomy around delicate structures such as the RLNs. Recent randomized trials, including REVATE [33, 34] and RAMIE [35], have provided evidence that RAMIE offers superior RLN preservation and more effective nodal dissection—especially in patients with SCC—compared to conventional thoracoscopic MIE. Although RAMIE may involve longer operative times or higher costs, its potential to improve oncologic clearance and functional outcomes justifies its increasing adoption, particularly in high‐volume centers with robotics expertise.

In parallel, mediastinoscopic esophagectomy has emerged as a promising alternative for patients with poor pulmonary function or those who are unsuitable for transthoracic approaches [8, 39, 40]. This technique offers notable advantages, including shorter operative times, avoidance of one‐lung ventilation, and reduced pulmonary complications. Robot‐assisted refinement of transhiatal and transcervical approaches—especially single‐port robotic systems, such as the da Vinci SP [61]—may further address some limitations of mediastinoscopic esophagectomy, including restricted working space and limited lymphadenectomy. However, the relatively high incidence of RLN paralysis and the inconsistent number of harvested lymph nodes across reports are of particular concern [8, 39]. Although mediastinoscopic esophagectomy will undoubtedly become one of the key pillars of MIE in cases where transthoracic approach is difficult and comprehensive reports are increasing, prospective multicenter studies are needed to further establish its oncological efficacy.

Despite the encouraging data for MIE, several issues remain unresolved. First, high‐quality evidence comes from selected high‐volume centers with significant experience in minimally invasive techniques, which may limit the generalizability of our findings. Second, heterogeneity in surgical techniques, patient selection, and tumor characteristics across studies complicate direct comparisons. In particular, differences in histological types, such as SCC and adenocarcinoma, and common sites of tumors in each region can lead to differences in surgical procedures and research results, making standardization difficult. Third, while short‐term outcomes have consistently favored MIE approaches, robust long‐term oncological data, especially for robotic and mediastinoscopic methods, remain limited.

Future research should focus on multicenter randomized trials with standardized protocols and adequate follow‐ups to evaluate the true oncological impact and cost‐effectiveness of each minimally invasive approach. Determining the appropriate indications for transthoracic versus robot‐assisted versus mediastinoscopic techniques based on tumor location, histology, and patient comorbidities is crucial for tailoring individualized treatment strategies. These researches will reveal the importance of the mediastinoscopic techniques, as well as other MIEs, in particularly for patients unfit for transthoracic procedures.

In conclusion, thoracoscopic, robot‐assisted, and mediastinoscopic esophagectomies are complementary options for MIE, each with unique advantages and limitations. With continued refinement, improved instrumentation, and accumulating evidence, MIE techniques are expected to further improve the safety and efficacy of esophageal cancer surgery.

## Author Contributions

**Hirotaka Konishi:** conceptualization, methodology, investigation, writing – original draft, writing – review and editing, validation. **Hiroyuki Inoue:** validation, investigation, writing – review and editing. **Hitoshi Fujiwara:** investigation, writing – review and editing, validation. **Atsushi Shiozaki:** conceptualization, writing – review and editing, project administration, supervision.

## Conflicts of Interest

The authors declare no conflicts of interest.
